# Supplementary material for: Heat adaptation of phage T7 under an extended genetic code
Source: Virus Evol. 2021 Dec 1;7(2):veab100. doi: 10.1093/ve/veab100 (PMC8923235; doi:10.1093/ve/veab100)
Supplement: veab100_Supp [file veab100_supp.zip › Sequencing_Analysis.zip!/Sequencing_Analysis/S1/evidence/SNP_9.html]

BRESEQ :: Evidence


| Predicted mutation | | | | | | |
| --- | --- | --- | --- | --- | --- | --- |
| evidence | seq id | position | mutation | annotation | gene | description |
| RA | NC\_001604.1 | 27,932 | T→C | intergenic (–/–) | – / – | –/– |

| Read alignment evidence... | | | | | | | | | | | |
| --- | --- | --- | --- | --- | --- | --- | --- | --- | --- | --- | --- |
|  | seq id | position | | ref | new | freq | score (cons/poly) | reads | annotation | genes | product |
| \* | NC\_001604.1 | 27,932 | 0 | T | C | 89.0% | 10366.0 / inf | 4378 | intergenic (–/–) | *–/–* | –/– |
| Reads supporting (aligned to +/- strand):  **ref** base T (199/277);  **new** base C (1850/1989);  **total** (2070/2308) | | | | | | | | | | | |
| Fisher's exact test for biased strand distribution *p*-value = 8.61e-03 | | | | | | | | | | | |
| Kolmogorov-Smirnov test that lower quality scores support variant *p*-value = 5.53e-01 | | | | | | | | | | | |

Only 100 of 4416 total aligned reads displayed.  

`GACTATCGGTGCGGTATTCCATGAAGAGTACACACGAGATGGTCAATTTCAGTTATTTACAATCACGAAAGGAGGATAACCATATGTGTTGGGCAGCCGCAATACCTAT‑‑‑‑‑‑‑‑‑‑‑‑CGCTATATCTGGCGCTCAGGCTATCAGTGGTCAGAACGCTCAGGCCAAAATGATTGCCGCTCAGACCGCTGCTGGTCGTCGTCAAGCTATGGAAATCATGAGGCAGACGAACATCCAGAATGCTGACCTATCGTTGCAAGCTCGAAGTAAACTTGAGGAAGCGTCCGCCGAGTTGACCTCACAGAACATGCAGAAGGTCCAAGCTATTGGGTCTATCCGAGCGGCTATCGGAGAGAGTATGCTTGAAGGTTCCTCAATGGACCGCATTAAGCGAGTCACAGAAGGACAGTTCATTCGGGAAGCCAATATGGTAACTGAGAACTATCGCCGTGACTACCAAGCAATCTTCGCACAGCAACTTGGTGGTACTCAAAGTGCTGCAAGTCAGATTGACGAAATCTATAAGAGCGAACAGAAACAGAAGAGTAAGCTACAGATGGTTCTGGACCCACTGGCTATCATGGGGTCTTCCGCTG  >  NC_001604.1/27645‑28229`  

                                                                                                                                                                                                                                                                                                           `|`                                                                                                                                                                                                                                                                                                           
`taagagacaggcggtattccatgaagagtacacacgagatggtcaatttcagttatttacaatcacgaaaggaggataaccatatgtgttgggcagccgcaatacctatcgctatatctggCGCTATATCTGGCGCTCAGGCTATCAGTGGTCAGAACGCTCAGGCCAAAATGATTGCCGCTCAGACCGCTGCTGGTCGTCGTCAAGCTATGGAAATCATGAGGCAGACGAACATCCAGAATGCTGACCTATCGTTGCAAGCTCGAAGTAAACTTGAGGAAGCGTCCGCCGAGTTGACCTc                                                                                                                                                                                                                                                                                                          <  1:561133‑M2/180‑1 (MQ=255)`  
`gactatcggtgcggtattccatgaagagtacacacgagatggtcaatttcagttatttacaatcacgaaaggaggataaccatatgtgttgggcagccgcaatacctatcgctatatctggCGCTATATCTGGCGCTCAGGCTATCAGTGGTCAGAACGCTCAGGCCAAAATGATTGCCGCTCAGACCGCTGCTGGTCGTCGTCAAGCTATGGAAATCATGAGGCAGACGAACATCCAGAATGCTGACCTATCGTTGCTAGCTCGAAGTAAACTTGAGGAAGCGTCAGCCGAGTTGACCTc                                                                                                                                                                                                                                                                                                          >  1:422916‑M2/122‑301 (MQ=255)`  
    `atcggtgcggtattccatgaagagtacacacgagatggtcaatttcagttatttacaatcacgaaaggaggataaccatatgtgttgggcagccgcaatacctatcgctatatctggCGCTATATCTGGCGCTCAGGCTATCAGTTGTCAGAACGCTCAGGCCAAAATGATTGCCGCTCAGACCGCTGCTGGTCGTCGTCAAGCTATGGAAATCATGAGGCAGACGAACATCCAGAATGCTTACCTATCGTTGCAAGCTCGAAGTAAACTTGAGGAAGCGTCCGCCGAGTTGACACCACAg                                                                                                                                                                                                                                                                                                      <  1:274394‑M2/184‑1 (MQ=255)`  
    `atcggtgcggtattccatgaagagtacacacgagatggtcaatttcagttatttacaatcacgaaaggaggataaccatatgtgttgggcagccgcaatacctatcgctatatctggCGCTATATCTGGCGCTCAGGCTATCAGTGGTCAGAACGCTCAGGCCAAAATGATTGCCGCTCAGACCGCTGCTGGTCGTCGTCAAGCTATGGAAATCATGAGGCAGACGAACATCCAGAATGCTGACCTATCGTTGCAAGCTCGAAGTAAACTTGAGGAAGCGTCCGCCGAGTTGACCCCACAg                                                                                                                                                                                                                                                                                                      <  1:399860‑M2/184‑1 (MQ=255)`  
             `gtattccatgaagagtacacacgagatggtcaatttcagttatttacaatcacgaaaggaggataaccatatgtgttgggcagccgcaatacctatcgctatatctggCGCTATATCTGGCGCTCAGGCTATCAGTGGTCAGAACGCTCAGGCCAAAATGATTGCCGCTCAGACCGCTGCTGGTCGTCGTCAAGCTATGGAAATCATGAGGCAGACGAACATCCAGAATGCTGACCTATCGTTGCAAGCTCGAAGTAAACTTGAGGAAGCGTCCGCCGAGTTGACCCCACAGAACATGCAg                                                                                                                                                                                                                                                                                             <  1:93211‑M2/193‑1 (MQ=255)`  
             `gtattccatgaagagtacacacgagatggtcaatttcagttatttacaatcacgaaaggaggataaccatatgtgttgggcagccgcaatacctatcgctatatctggCGCTATATCTGGCGCTCAGGCTATCAGTGGTCAGAACGCTCAGGCCAAAATGATTGCCGCTCAGACCGCTGCTGGTCGTCGTCAAGCTATGGAAATCATGAGGCAGACGAACATCCAGAATGCTGACCTATCGTTGCAAGCTCGAAGTAAACTTGAGGAAGCGTCCGCCGAGTTGACCCCACAGAACATGCAg                                                                                                                                                                                                                                                                                             >  1:227449‑M2/109‑301 (MQ=255)`  
               `attccatgaagagtacacacgagatggtcaatttcagttatttacaatcacgaaaggaggataaccatatgtgttgggcagccgcaatacctatcgctatatctggCGCTATATCTGGCGCTCAGGCTATCAGTGGTCAGAACGCTCAGGCCAAAATGATTGCCGCTCAGACCGCTGCTGGTCGTCGTCAAGCTATGGAAATCATGAGGCAGACGAACATCCAGAATGCTGACCTATCGTTGCAAGCTCGAAGTAAACTTGAGGAAGCGTCCGCCGAGTTGACCCCACAGAACATGCAGaa                                                                                                                                                                                                                                                                                           >  1:573449‑M2/107‑301 (MQ=255)`  
                 `tccatgaagagtacacacgagatggtcaatttcagttatttacaatcacgaaaggaggataatcatatgtgttgggcagccgcaatacctatcgctatatctggCGCTATATCTGGCGCTCAGGCTATCAGTGGTCAGAACGCTCAGGCCAAAATGATTGCCGCTCAGACTGCTGCTGGTAGTTGTCAAGCTATGGAAATCATGAGGCAGAAGAACATCCAGAATGCTGACCTATCGTTGCAAGCTCGAAGTAAACTTGAGGAAGCGTCCGCAGAGTTTACCTCAAAGAACATGACGAAgg                                                                                                                                                                                                                                                                                         >  1:66646‑M2/105‑301 (MQ=255)`  
                 `tccatgaagagtacacacgagatggtcaatttcagttatttacaatcacgaaaggaggataaccatatgtgttgggcagccgcaatacctatcgctatatctggCGCTATATCTGGCGCTCAGGCTATCAGTGGTCAGAACGCTCAGGCCAAAATGATTGCCGCTCAGACCGCTGCTGGTCGTCGTCAAGCTATGGAAATCATGAGGCAGACGAACATCCAGAATGCTGACCTATCGTTGCAAGCTCGAAGTAAACTTGAGGAAGCGTCCGCCGAGTTGACCCCACAGAACATGCAGAAgg                                                                                                                                                                                                                                                                                         >  1:6787‑M2/105‑301 (MQ=255)`  
                 `tccatgaagagtacacacgagatggtcaatttcagttatttacaatcacgaaaggaggataaccatatgtgttgggcagccgcaatacctatcgctatatctggCGCTATATCTGGCGCTCAGGCTATCAGTGGTCAGAACGCTCAGGCCAAAATGATTGCCGCTCAGACCGCTGCTGGTCGTCGTCAAGCTATGGAAATCATGAGGCAGACGAACATCCAGAATGCTGACCTATCGTTGCAAGCTCGAAGTAAACTTGAGGAAGCGTCCGCCGAGTTGACCCCACAGAACATGCAGAAgg                                                                                                                                                                                                                                                                                         >  1:325569‑M2/105‑301 (MQ=255)`  
                 `tacatgaagagtacacacgagatggtcaatttcagttatttacaatcacgaaaggaggataaccatatgtgttgggcagccgcaatacctatcgctatatctggCGCTATATCTGGCGCTCAGGCTATCAGTGGTCAGAACGCTCAGGCCAAAATGATTGCCGCTCAGACCGCTGCTGGTCGTCGTCAAGCTATGGAAATCATGAGGCAGACGAACATCCAGAATGCTGACCTATCGTTGCAAGCTCGAAGTAAACTTTAGGAAGCGTCCGCCGAGTTGACCCCACAGAACATGCAGAAgg                                                                                                                                                                                                                                                                                         <  1:35597‑M2/197‑1 (MQ=255)`  
                  `ccatgaagagtacacacgagatggtcaatttcagttatttacaatcacgaaaggaggataaccatatgtgttgggcagccgcaatacctatcgctatatctggCGCTATATCTGGCGCTCAGGCTATCAGTGGTCAGAACGCTCAGGCCAAAATGATTGCCGCTCAGACCGCTGCTGGTCGTCGTCAAGCTATGGAAATCATGAGGCAGACGAACATCCAGAATGCTGACCTATCGTTGCAAGCTCGAAGTAAACTTGAGGAAGCGTCCGCCGAGTTGACCCCAcagaacctgtctcttat                                                                                                                                                                                                                                                                                        >  1:51515‑M2/104‑293 (MQ=255)`  
                   `catgaagagtacacacgagatggtcaatttcagttatttacaatcacgaaaggaggataaccatatgtgttgggcatccgcaatacctatcgctatatctggCGCTATATCTGGCGCTCAGGCTATCAGTGGTCAGAACGCTCAGGCCAAAATGATTGCCGCTCAGACCGCTGCTGGTCGTCGTCAAGCTATGGAAATCATGAGGCAGACGAACATCCAGAATGCTGACCTATCGTTGCAAGCTCGAAGTAAACTTGAGGAAGCGTCCGCCGAGTTGACACCACAGAACATGCAGAAGGTc                                                                                                                                                                                                                                                                                       >  1:60089‑M2/103‑301 (MQ=255)`  
                        `agagtacacacgagatggtcaatttcagttatttacaatcacgaaaggaggataaccagaggtgttgggcagccgaaatacctatcgctatatctggCGCTATATCTGGCGCTCAGGCTATCAGTGGTCAGAACGCTCAGGCCAAAATGATTGCCGCTCAGACCGCTGCTGGTCGTCGTCAAGCTATGGAAATCATGAGGCAGACGAACATCCAGAATGCTGACCTATCGTTGCAAGCTCGAAGTAAACTTGAGGAAGCGTCCGCCGAGTTGACCCCACAGAACATGCAGAAGGTCCAAGc                                                                                                                                                                                                                                                                                  <  1:208267‑M2/204‑1 (MQ=255)`  
                         `gagtacacacgagatggtcaatttcagttatttacaatcacgaaaggaggataaccatatgtgttgggcagccgcaatacctatcgctatatctggCGCTATATCTGGCGCTCAGGCTATCAGTGGTCAGAACGCTCGGGCCAAAATGATTGCCGCTCAGACCGCTGCTGGTCGTCGTCAAGCTATGGAAATCATGAGGCAGACGAACATCCAGAATGCTGACCTATCGTTGCAAGCTCGAAGTAAACTTGAGGAAGCGTCCGCCGAGTTGACCCCACAGAACATGCAGAAGGTCCAAgcc                                                                                                                                                                                                                                                                                 >  1:285946‑M2/97‑300 (MQ=255)`  
                           `gtacacacgagatggtcaatttcagttatttacaatcacgaaaggaggataaccatatgtgttgggcagccgcaatacctatcgctatatctggCGCTATATCTGGCGCTCAGGCTATCAGTGGTCAGAACGCTCAGGCCAAAATGATTGCCGCTCAGACCGCTGCTGGTCGTCGTCAAGCTATGGAAATCATGAGGCAGACGAACATCCAGAATGCTGACCTATCGTTGCAAGCTCGAAGTAAACTTGAGGAAGCGTCCGCCGAGTTGACCCCACAGAACATGCAGAAGGTCCaagcctt                                                                                                                                                                                                                                                                               >  1:473054‑M2/95‑298 (MQ=255)`  
                           `gtacacacgagatggtcaatttcagttatttacaatcacgaaaggaggataaccatatgtgttgggcagccgcaatacccatcgctatatctggCGCTATATCTGGCGCTCAGGCTGTCAGTGGTCAGAACGCTCAGGCCAAAATGATTGCCGCTCAGACAGCTGCTGGTCGTCGCCAAGCTATGGAAATCATGAGGCAGACGAAGACCAAGAATGCTGACCTACCGTTGCACGATCGAAGTAAACTTGAGGACGCGACCCCCGTGTTGACCACACAGAACATGcagagggtctgaactat                                                                                                                                                                                                                                                                               >  1:525406‑M2/95‑293 (MQ=255)`  
                            `gtataagagacaggtcaatttcagttatttacaatcacgaaaggaggataaccatatgtgttgggcagccgcaatacctatcgctatatctggCGCTATATCTGGCGCTCAGGCTATCAGTGGTCAGAACGCTCAGGCCAAAATGATTGCCGCTCAGACCGCTGCTGGTCGTCGTCAAGCTATGGAAATCATGAGGCAGACGAACATCCAGAATGCTGACCTATCGTTGCAAGCTCGAAGTAAACTTGAGGAAGCGTCCGCCGAGTTGACCTCACAGAACATGCAGAAGGTCCAagctctg                                                                                                                                                                                                                                                                              <  1:435151‑M2/208‑4 (MQ=255)`  
                               `tataagagacagcaatttcagttatttacaatcacgaaaggaggataaccatatgtgttgggcagccgcaatacctatcgctatatctggCGCTATATCTGGCGATCAGGCTATCAGTGGTTAGAACGCTCAGGCCAAAATGATTGCCGCTCAGACCGCTGCTGGTCGTCGTCAAGCTATGGAAATCATGAGGCAGACGAACATCCAGAATGCTGACCTATCGTTGCAAGCTCGAAGTAAACTTGAGGAAGCGTCCGCCGAGTTGACCCCACAGAACATGCAGAAGGTCCAAGCTATTggg                                                                                                                                                                                                                                                                           <  1:332129‑M2/211‑1 (MQ=255)`  
                                `cacgagatggtcaatttcagttatttacaatcacgaaaggaggataaccatatgtgttgggcagccgcaatacctatcgctatatctggCGCTATATCTGGCGCTCAGGCTATCAGTGGTCAGAACGCTCAGGCCAAAATGATTGCCGCTCAGACCGCTGCTGGTCGTCGTCAAGCTATGGAAATCATGAGGCAGACGAACATCCAGAATGCTGACCTATCGTTGCAAGCTCGAAGTAAACTTGAGGAAGCGTCCGCCGAGTTGACCCCACAGAACATGCAGAAGGTCCAAGCTATTGGGt                                                                                                                                                                                                                                                                          <  1:232249‑M2/212‑1 (MQ=255)`  
                                     `atgtgtataagagacagatttacaatcacgaaaggaggataaccatatgtgttgggcagccgcaatacctatcgctatatctggCGCTATATCTGGCGCTCAGGCTATCAGTGGTCAGAACGCTCAGGCCAAAATGATTGCCGCTCAGACCGATGCTGGTCGTTGTCAAGCTATGGAAATCATGAGGCAGACGAACATCCAGAATGCTGACCTATCGTTGCAAGCTCGAAGTAAACTTGAGGAAGCGTCCGCCGAGTTGACCCCACAGAACATGCAGAAGGTCCAAGCTATTGGGTCTATc                                                                                                                                                                                                                                                                     <  1:291969‑M2/217‑1 (MQ=255)`  
                                      `atggtcaatttcagttatttacaatcacgaaaggaggataaccatatgtgttgggcagccgcaatacctatcgctatatctggCGCTATATCTGGCGCTCAGGCTATCAGTGGTCAGAACGCTCAGGCCAAAATGATTGCCGCTCAGACCGCTGCTGGTCGTCGTCAAGCTATGGAAATCATGAGGCAGACGAACATCCAGAATGCTGACCTATCGTTGCAAGCTCGAAGTAAACTTGAGGAAGCGTCCGCCGAGTTGACCTAACAGAACATGCAGAAGGTCCAAGCTATtgggtcctgtc                                                                                                                                                                                                                                                                    >  1:519862‑M2/84‑296 (MQ=255)`  
                                       `aggttaattttagttatttacaatcacgaaaggaggataaccatatgtgttgggcagccgcaatacctatcgctatatctggCGCTATATCTGGCGCTCAGGCTATCAGTGGTCAGAACGCTCAGGCCAAAATGATTGCCGCTCAGACCGCTGCTGGTCGTCGTCAAGCTATGGAAATCATGAGGCAGACGAACATCCAGAATGCTGGCCTATCGTTGCAAGCTCGAAGTAAACTTGAGGAAGCGTCCGCCGAGTTGACCCCACAGAACATGCAGAAGGTCCAAGCTATTGGGTCTATCCg                                                                                                                                                                                                                                                                   <  1:480721‑M2/219‑1 (MQ=255)`  
                                                     `tatttacaatcacgaaaggaggataaccatatgtgttgggcagccgcaatacctatcgctatatctggCGCTATATCTGGCGCTCAGGCTATCAGTGGTCAGAACGCTCAGGCCAAAATGATTGCCGCTCAGACCGCTGCTGGTCGTCGTCAAGCTATGGAAATCATGAGGCAGACGAACATCCAGAATGCTGACCTATCGTTGCAAGCTCGAAGTAAACTTGAGGAAGCGTCCGCCGAGTTGACCCCACAGAACATGCAGAAGGTCCAAGCTATTGGGTCTATCCGAGCGGCTATCGgag                                                                                                                                                                                                                                                     <  1:542674‑M2/233‑1 (MQ=255)`  
                                                       `tttacaatcacgaaaggaggataaccatatgtgttgggcagccgcaatacctatcgctatatctggCGCTATATCTGGCGCTCAGGCTATCAGTGGTCAGAACGCTCAGGCCAAAATGATTGCCGCTCAGACCGCTGCTGGTCGTCGTCAAGCTATGGAAATCATGAGGCAGACGAACATCCAGAATGCTGACCTATCGTTGCAAGCTCGAAGTAAACTTGAGGAAGCGTCCGCCGAGTTGACCCCACGGAACATGCAGAAGGTCCAAGCTATTGGGTCTATCCGAGCGGCTATCGgagag                                                                                                                                                                                                                                                   <  1:150051‑M2/235‑1 (MQ=255)`  
                                                       `tttacaatcacgaaaggaggataaccatatgtgttgggcagccgcaatacctatcgctatatctggCGCTATATCTGGCGCTCAGGCTATCAGTGGTCAGAACGCTCAGGCCAAAATGATTGCCGCTCAGACCGCTGCTGGTCGTCGTCAAGCTATGGAAATCATGAGGCAGACGAACATCCAGAATGCTGACCTATCGTTGCAAGCTCGAAGTAAACTTGAGGAAGCGTCCGCCGAGTTGACCCCACAGAACATGCAGAAGGTCCAAGCTATTGGGTCTATCCGAGCGGCTATCGgagag                                                                                                                                                                                                                                                   <  1:486398‑M2/235‑1 (MQ=255)`  
                                                       `tttacaatcacgaaaggaggataaccatatgtgttgggcagcagcaatacctatcgctatatctggCGCTATATCTGGCGCTCAGGCTATCAGTGGTCAGAACGCTCAGGCCAAAATGATTGCCGCTCAGACCGCTGCTGGTAGTCGTCAAGCTATGGAAATCATGAGGCAGACGAACATCCAGAATGCTGACCTATCGTTACAAGCTCGAAGTAAACTTGAGGAAGCGTCCGCCGAGTTGACCCCACAGAACATGCAGAAGGTCCAAGCTATTGGGTCTATCCGAGCGGCTATCGgagag                                                                                                                                                                                                                                                   <  1:137409‑M2/235‑1 (MQ=255)`  
                                                       `tttacaagcacgaaaggaggataaccatatgtgttgggcagccgcaataccgatcgctatatctggCGCTATATCTGGCGCTCAGGCTATCAGTGGTCAGAACGCTCAGGCCAAAATGATTGCCGCTCAGACCGCTGCTGGTCGTCGTCAAGCTATGGAAATCATGAGGCAGACGAACATCCAGAATGCTGACCTATCGTTGCAAGCTCGAAGTAAACTTGAGGAAGCGTCCGCCGAGTTGACCCCACAGAACATGCAGAAGGTCCAAGCTATTGGGTCTATCCGAGCGGCTATCGgagag                                                                                                                                                                                                                                                   <  1:311677‑M2/235‑1 (MQ=255)`  
                                                               `cacgaaaggaggataaccatatgtgttgggcagccgcaatacctatcgctatatctggCGCTATATCTGGCGCTCAGGCTATCAGTGGTCAGAACGCTCAGGCCAAAATGATTGCCGCTCAGACCGCTGCTGGTCGTCGTCAAGCTATGGAAATCATGAGGCAGACGAACATCCAGAATGCTGACCTATCGTTGCAAGCTCGAAGTAAACTTGAGGAAGCGTCCGCCGAGTTGACCCCACAGAACATGCAGAAGGTCCACGCTATTGGGTCTATCCTAGCGGCTATCGGAGAGAGTATGCt                                                                                                                                                                                                                                           >  1:467533‑M2/59‑301 (MQ=255)`  
                                                               `cacgaaaggaggataaccatatgtgttgggcagccgcaatacctatcgctatatctggCGCTATATCTGGCGCTCAGGCTATCAGTGGTCAGAACGCTCAGGCCAAAATGATTGCCGCTCAGACCGCTGCTGGTCGTCGTCAAGCTATGGAAATCATGAGGCAGACGAACATCCAGAATGCTGACCTATCGTTGCAAGCTCGAAGTAAACTTGAGGAAGCGTCCGCCGAGTTGACCCCACAGAACATGCAGAAGGTCCAAGCTATTGGGTCTACCCGAGCGGCTATCGGAGAGGGTATGCt                                                                                                                                                                                                                                           >  1:59504‑M2/59‑301 (MQ=255)`  
                                                               `cACGAAAGGAGGATAACCATATGTGTTTGGCAGCCGCAATACCTATCGCTATATCTGCCGCTATATCTGGCGCTCAGGCTATTAGTGTTCAGAACGCTCAGGCCAAAATGATTGCCGCTCAGACCTCTGCTGGTCGTCGTCAAGCTATTGAATTCATGAGGCACACGTACTTCCAGATTGCTTTCCTATCGTTTCATGCTCGTAGTATTCTTTTGGAATCGTCTCCCGAGTTGTCCCCTCAGATCATGCAGTAGTTCCACCCTTTTTGTTCTATCCGaccttctttcggtgtgtgtttgtt                                                                                                                                                                                                                                           >  1:261197/1‑278 (MQ=255)`  
                                                                     `aggaggataaccatatgtgttgggcagccgcaatacctatcgctatatctggCGCTATATCTGGCGCTCAGGCTATCAGTGGTCAGAACGCTCAGGCCAAAATGATTGCCGCTCAGACCGCTGCTGGTCGTCGTCAAGCTATGGAAATCATGAGGCAGACGAACATCCAGAATGCTGACCTATCGTTGCAAGCTCGAAGTAAACTTGAGGAAGCGTCCGACGAGTTGACCCCACAGAACATGCAGAAGGTCCAAGCTATTGGGTCTATCCGAGCGGCTATCGGAGAGAGTATGCTTGAAgg                                                                                                                                                                                                                                     <  1:255282‑M2/249‑1 (MQ=255)`  
                                                                       `gaggataaccatatgtgttgggcagccgcaatacctatcgctatatctggCGCTATATCTGGCGCTCAGGCTATCAGTGGTCAGAACGCTCAGGCCAAAATGATTGCCGCTCAGACCGCTGCTGGTCGTCGTCAAGCTATGGAAATCATGAGGCAGACGCACATCCAGAATGCTGACCTATCGTTGCAAGCTCGAAGTAAACTTGAGGAAGCGTCCGCCGAGTTGACCCCACAGAACATGCAGAAGGTCCAAGCTATTGGGTCTATCCGAGCGGCTATCGGAGAgaggatgctctgtctct                                                                                                                                                                                                                                   >  1:362244‑M2/51‑293 (MQ=255)`  
                                                                       `gaggataaccatatgtgttgggcagccgcaatacctatcgctatatctggCGCTATATCTGGCGCTCAGGCTATCAGTGGTCAGAACGCTCAGGCCAAAATGATTGCCGCTCAGACCGCTGCTGGTCGTCGTCAAGCTATGGAAATCATGAGGCAGACGAACATCCAGAATGCTGACCTATCGTTGCAAGCTCGAAGTAAACTTGAGGAAGCGTCCGCCGAGTTGACCCCACAGAACATGCAGAAGGTCCAAGCTATTGGGTCTATCCGAGCGGCTATCGGAGAGAGTATGCTTGAAGGtt                                                                                                                                                                                                                                   >  1:185603‑M2/51‑301 (MQ=255)`  
                                                                           `agaaccatctgtgttgggcatccgcaatacctttcgctatatctggCGCTATATCTGGCGCTCAGGCTATCAGTAGTCAGAACGCTCAGGCCAAAATGATTGCCGCTCAGACCGCTGCTGGTCTTCGTCTAGCTATGTAAATCATGAGGCAGACGAACATCCAGAATGCTTACCTATCGTTGCAAGCTCGAAGTAAACTTGAGGAAGCGTCCGCCGAGTTGACCCCACAGAACATGCAGAAGGTCCAAGCTATTGGGTCTATCCGAGCGGCTATCGGAGAGAGTATGCTTGAAGGTTCCTc                                                                                                                                                                                                                               <  1:162256‑M2/255‑1 (MQ=255)`  
                                                                               `ccatatgtgttgggcagccgcaatacctatcgctatatctggCGCTATATCTGGCGCTCAGGCTATCAGTGGTCAGAACGCTCAGGCCAAAATGATTGCCGCTCAGACCGCTGCTGGTCGTCGTCAAGCTATGGAAATCATGAGGCAGACGAACATCCAGAATGCTGACCTATCGTTGCAAGCTCGAAGTAAAATTGAGGAAGCGTCCGCCGAGTTGACCCCACAGAACATGCAGAAGGTCCAAGCTATTGGGTCTATCCGAGCGGCTATCGGAGAGAGtatgcttgaaggctgtctctta                                                                                                                                                                                                                           >  1:238575‑M2/43‑291 (MQ=255)`  
                                                                               `ccatatgtgttgggcagccgcaatacctatcgctatatctggCGCTATATCTGGCGATCAGGCTATCAGTGGTCAGAACGCTCAGGCCAAAATGATTGCCGCTCAGACCGCTGCTGGTCGTCGTCAAGCTATGGAAATCATGAGGCAGACGAACATCCAGAATGCTGACCTATCGTTGCAAGCTCGAAGTAAACTTGAGGAAGCGTCCGCCGAGTTGACCCCACAGAACATGCAGAAGGTCCAAGCTATTGGGTCTATCCGAGCGGCTATCGGAGAGAGTATGCTTGAAGGTTCCTCAATg                                                                                                                                                                                                                           >  1:245159‑M2/43‑301 (MQ=255)`  
                                                                                `catatgtgttgggcagccgcaatacctatcgctatatctggCGCTATATCTGGCGCTCAGGCTATCAGTGGTCAGAACGCTCAGGCCAAAATGATTGCCGCTCAGACCGCTGCTGGTCGTCGTCAAGCTATGGAAATCATGAGGCAGACGAACATCCAGAATGCTGACCTATCGTTGCAAGCTTGAAGTAAACTTGAGGAAGCGTCCGCCGAGTTGACCCCACAGAACATGCAGAAGGTCCAAGCTATTGGGTCTATCCGAGCGGCTATCGGAGAGAGTATGCTTGAAGGTTCCTCAATgg                                                                                                                                                                                                                          <  1:548165‑M2/260‑1 (MQ=255)`  
                                                                                `cataggtgtttggcagccgcaatacctatcgctatatctggCGCTAGATCTGGCGCTCAGGCTATCAGTGGTCAGAACGCTCAGGCCAAAATGATTGCCGCTCAGACCGCTGCTGGTCGTCGTCAAGCTATGGAAATCATGAGGCAGACGAACATCCAGAATGCTGACCTATCGTTGCAAGCTCGAAGTAAACTTGAGGAAGCGTCCGCCGAGTTGACCCCACAGAACATGCAGAAGGTCCAAGCTATTGGGTCTATCCGAGCGGCTATCGGAGAGAGTATGCTTGAAGGTTCCTCAATgg                                                                                                                                                                                                                          <  1:581384‑M2/260‑1 (MQ=255)`  
                                                                                   `atgtgttgggcagccgcaatacctatcgctatatctggCGCTATATCTGGCGCTCAGGCTATCAGTGGTCAGAACGCTCAGGCCAAAATGATTGCCGCTCAGACCGCTGCTGGTCGTCGTCAAGCTATGGAAATCATGAGGCAGACGAACATCCAGAATGCTGACCTATCGTTGCAAGCTCGAAGTAAACTTGAGGAAGCGTCCGCCGAGTTGACCTCACAGAACATGCAGAAGGTCCAAGCTATTGGGTCTATCCGAGCGGCTATCGGAGAGAGTATGCTTGAaggttcctcctgtctct                                                                                                                                                                                                                       >  1:370178‑M2/39‑293 (MQ=255)`  
                                                                                     `gtgttgggcagccgcaatacctatcgctatatctggCGCTATATCTGGCGATCAGGCTATCAGTGGTCAGAACGCTCAGGCCAAAATGATTGCCGCTCAGACCGCTGCTGGTCGTCGTCAAGCTATGGAAATCATGAGGCAGACGAACATCCAGAATGCTGACCTATCGTTGCAAGCTCGAAGTAAACTTGAGAAAGCGTCCGCAGCGTTGACCTCACAGAACATGAAGAAGGTCCAAGCTATTGGGTCTATCCGAGCGGCTATGGGGAAGAGTACTCTTTAaggttcctcactctgtctt                                                                                                                                                                                                                     >  1:376857‑M2/37‑292 (MQ=255)`  
                                                                                           `ggcagccgcaatacctatcgctatatctggCGCTATATCTGGCGCTCAGGCTATCAGTGGTCAGAACGCTCAGGCCAAAATGATTGCCGCTCAGACCGCTGCTGGTCGTCGTCAAGCTATGGAAATCATGAGGCAGACGAACATCCAGAATGCTGACCTATCGTTGCAAGCTCGAAGTAAACTTGAGGAAGCGTCCGCCGAGTTGACCCCACAGAACATGCAGAAGGTCCAAGCTATTGGGTCTATCCGAGCGGCTATCGGAGAGAGTATGCTTGAAGGTTCCTCAATGGACCGCATTAAg                                                                                                                                                                                                               <  1:174201‑M2/271‑1 (MQ=255)`  
                                                                                             `tgtgtataagcgacagcgctatatctgtCTCTATATCTGGCGCTCAGGCTATCAGTGGTCAGAACTCTCAGTCCAAAATGATTGCCACTCAGACCGCTGCTGGTCGTCGTCAAGCTACGGAAATCATGAGGCAGACGAACATCCAGAATGCTGCCCTATCGTTGCAAGCTCGAAGTAAACTTGAGGAAGCGTCCGCCGAGTTGACCCCACAGAACATGCAGAAGGTCCAAGCTATTGGGTCTATCCGAGCGGCTATCGGAGAGAGTATGCTTGAAGGTTCCTCAATGGACCGCATTAAGCg                                                                                                                                                                                                             <  1:531422‑M2/273‑1 (MQ=255)`  
                                                                                             `cagctgcaatacctatcgctatatctggCGCTATAGCTGGCGCTCAGGCTAACAGTGGTCAGAACGCTCAGGCCAAAATGATTGCCGCTCAGACCGCTGCTGGTCGTCGTCAAGCTATGGAAATCATGAGGCAGACGAACATCCAGAATGCTGACCTATCGTTGCAAGCTCGAAGTAAACTTGAGGAAGCGTCCGCCGAGTTGACCCCACAGAACATGCAGAAGGTCCAAGCTATTGGGTCTATCCGAGCGGCTATCGGAGAGAGTATGCTTGAAGGTTCCTCAATGGACCGCATTAAGCg                                                                                                                                                                                                             <  1:442565‑M2/273‑1 (MQ=255)`  
                                                                                               `gccgcaatacctatcgctatatctggCGCTATATCTGGCGCTCAGGCTATCAGTGGTCAGAACGCTCAGGCCAAAATGATTGCCGCTCAGACCGCTGCTGGTCGTCGTCAAGCTATGGAAATCATGAGGCAGACGAACATCCAGAATGCTGACCTATCGTTGCAAGCTCGAAGTAAACTTGAGGAAGCGTCCGCCGAGTTGACCCCACAGAAAATGCAGAAGGTCCAAGCTATTGGGTCTATCCGAGCGGCTATCGGAGAGAGTATGCTTGAAGGTTCCTCAATGGACCGCATTAagagag                                                                                                                                                                                                           >  1:394171‑M2/27‑301 (MQ=255)`  
                                                                                                      `tacctatcgctatatctggCGCTATATCTGGCGCTCAGGCTATCAGTGGTCAGAACGCTCAGGCCAAAATGATTGCCGCTCAGACCGCTGCTGGTCGTCGTCAAGCTATGGAAATCATGAGGCAGACGAACATCCAGAATGCTGACCTATCGTTGCAAGCTCGAAGTAAACTTGAGGAAGCGTCCGCCGAGTTGACCCCACAGAACATGCAGAAGGTCCAAGCTATTGGGTCTATCCGAGCGGCTATCGGAGAGAGTATGCTTGAAGGTTCCTCAATGGACCGCATTAAGCGAGTCACAGa                                                                                                                                                                                                    <  1:448868‑M2/282‑1 (MQ=255)`  
                                                                                                       `acctatcgctatatctggCGCTATATCTGGCGCTCAGGCTATCAGTGGTCAGAACGCTCAGGCCAAAATGATTGCCGCTCAGACCGCTGCTGGTCGTCGTCAAGCTATGGAAATCATGAGGCAGACGAACATCCAGAATGCTGACCTATCGTTGCAAGCTCGAAGTAAACTTGAGGAAGCGTCCGCCGAGTTGACCTCACAGAACATGCAGAAGGTCCAAGCTATTGGGTCTATCCGAGCGGCTATCGGAGAGAGTATGCTTGAAGGTTCCTCAATGGACCGCATTAAGCGAGGCAAAGaa                                                                                                                                                                                                   >  1:55424‑M2/19‑301 (MQ=255)`  
                                                                                                        `cctatcgctatatctggCGCTATATCTGGCGCTCAGGCTATCAGTGGTCAGAACGCTCAGGCCAAAATGATTGCCGCTCAGACCGCTGCTGGTCGTCGTCAAGCTATGGAAATCATGAGGCAGACGAACATCCAGCATGCTGACCTATCGTTGCAAGCTCGCAGTAAACTTGAGGAAGCGTCCGCCGAGTTGACCCAACAGAACATGCAGAAGGTCCAAGCTATAGGGTCTATCCGAGCGGCTATCGGAGAGAGTGTGCTTGAAGGTTCATCAATGGACCGCATTCAGCGAGTCACTGAAg                                                                                                                                                                                                  >  1:124417‑M2/18‑301 (MQ=255)`  
                                                                                                         `ctatcgctatatctggCGCTATATCTGGCGCTCAGGCTATCAGTGGTCAGAACGCTCAGGCCAAAATGATTGCCGCTCAGACCGCTGCTGGTCGTCGTCAAGCTATGGAAATCATGAGGCAGACGAACATCCAGAATGCTGACCTATCGATGCAAGCTCGAAGTAAACTTGAGGAAGCGTCCGCCGAGTTGACCCCACAGAACATGCAGAAGGTCCAAGCTATTGGGTCTATCCGAGCGGCTATCGGAGAGAGTATGCTTGAAGGTTCCTCAATGGACCGCATTAAGCGAGTCACAGAAgg                                                                                                                                                                                                 >  1:621665‑M2/17‑301 (MQ=255)`  
                                                                                                           `gtcgctatatctggTGCTATATCTGGCGCTCAGGCTATCAGTGGTCAGAACGCTCAGGCCAAAATGATTGCCGCTCAGACCGCTGCTGGTCGTCGTCAAGCTATGGAAATCATGAGGCAGACGAACATCCAGAATGCTGACCTATCGTTGCAAGCTCGAAGTAAACTTGAGGAAGCGTCCGCCGAGTTGACCCCACAGAACATGCAGAAGGTCCAAGCTATTGGGTCTATCCGAGCGGTTATCGGAGAGAGTATGCTTGAAGGTTCCTCAATGGACCGCATTAAGCGAGTCACAGAAGGAc                                                                                                                                                                                               <  1:416905‑M2/287‑1 (MQ=255)`  
                                                                                                           `atcgctatatctggCGCTATATCTGGCGCTCAGGCTATCAGTGGTCAGAACGCTCAGGCCAAAATGATTGCCGCTCAGACCGCTGCTGGTCGTCGTCAAGCTATGGAAATCATGAGGCAGACGAACATCCAGAATGCTGACCTATCGTTGCAAGCTCGAAGTAAACTTGAGGAAGCGTCCGCCGAGTTGACCCCACAGAACATGCAGAAGGTCCAAGCTATTGGGTCTATCCGAGCGGCTATCGGAGAGAGTATGCTTGAAGGTTCCTCAATGGACCGCATTAAGCGAGTCACAGAAGGAc                                                                                                                                                                                               <  1:42889‑M2/287‑1 (MQ=255)`  
                                                                                                                   `atctggCGCTATATCTGGCGCTCAGGCTATCAGTTGTCAGAACGCTCAGGCCAAAATGATTGCCGCTCAGACCGCTGCTGGTCGTCGTCAAGCTATGGAAATCATGAGGCAGACGAACATCCAGAATGCTGACCTATCGTTGCAAGCTCGAAGTAAACTTGAGGAAGCGTCCGCCGAGTTGACCCCACAGAACATGCAGAAGGTCCAAGCTATTGGGTCTATCCGAGCGGCTATCGGAGAGAGTATGCTTGAAGGTTCCTCAATGGACCGCATTAAGCGAGTCACAGAAGGACAGTTCAtt                                                                                                                                                                                       >  1:119098‑M2/7‑301 (MQ=255)`  
                                                                                                                   `atctggCGCTATATCTGGCGCTCAGGCTATCAGTGGTCAGAACGCTCAGGCCAAAATGATTGCCGCTCAGACCGCTGCTGGTCGTCGTCACGCTATGGAAATCATGAGGCAGACGAACATCCAGAATGCTTACCTATCGTTGCAAGCTCGAAGTAAACTTGAGGAACCGTCCGCCGAGTTGACCCCACAGAACATGCAGAAGGTCCAAGCTATTGGGTCTATCCGAGCGGCTATCGGAGAGACTATGCTTGAAGGTTCCTCAACTGCCCGCATTATGCGAGTCACAGATGGACAGTGCAtt                                                                                                                                                                                       >  1:455504‑M2/7‑301 (MQ=255)`  
                                                                                                                   `atctggCGCTATATCTGGCGCTCAGGCTATCAGTGGTCAGAACGCTCAGGCCAAAATGATTGCCGCTCAGACCGCTGCTGGTCGTCGTCAAGCTATGGAAATCATGAGGCAGACGAACATCCAGAATGCTGACCTATCGTTGCAAGCTCGAAGTAAACTTGAGGAAGCGTCCGCCGAGTTGACCCCACAGAACATGCAGAAGGTCCAAGCTATTGGGTCTATCCGAGCGGCTATCGGAGAGAGTATGCTTGAAGGTTCCTCAATGGACCGCATTAAGCGAGTCACAGAAGGACAGTTCAtt                                                                                                                                                                                       >  1:568323‑M2/7‑301 (MQ=255)`  
                                                                                                                       `ggCGCTATATCTGGCGCTCAGGCTATCAGTGGTCAGAACGCTCAGGCCAAAATGATTGCCGCTCAGACCGCTGCTGGTCGTCGTCAAGCTATGGAAATCATGAGGCAGACGAACATCCAGAATGCTGACCTATCGTTGCAAGCTCGAAGTAAACTTGAGGAAGCGTCCGCCGAGTTGACCCCACAGAACATGCAGAAGGTCCAAGCTATTGGGTCTATCCGAGCGGCTATCGGAACGAGTATGCTTGAAGGTTCCTCAATGGACCGCATTAAGCGAGTCACAGAAGGACCGTTCATTCggg                                                                                                                                                                                   >  1:461953‑M2/3‑301 (MQ=255)`  
                                                                                                                             `atatCTGGCGCTCAGGCTATCAGTGGTCAGAACGCTCAGGCCAAAATGATTGCCGCTCAGACCGCTGCTGGTCGTCGTCAAGCTATGGAAATCATGAGGCAGACGAACATCCAGAATGCTGACCTATCGTTGCAAGCTCGAAGTAAACTTGAGGAAGCGTCCGCCGAGTTGACCTCACAGAACATGCAGAAGGTCCAAGCTATTGGGTCTATCCGAGCGGCTATCGGAGAGAGTATGCTTGAAGGTTCCTCAATGGACCGCATTAAGCGAGTCCCAGAAGGACAGTTCATTCGGGAAGCCa                                                                                                                                                                             >  1:626450/1‑301 (MQ=255)`  
                                                                                                                                   `ggCGCTCAGGCTATCAGTGGTCAGAACGCTCAGGCCAAAATGATTGCCGCTCAGACCGCTGCTGGTCGTCGTCAAGCTATGGAAATCATGAGGCAGACGAACATCCAGAATGCTGACCTATCGTTGCAAGCTCGAAGTAAACTTGAGGAAGCGTCCGCCGAGTTGACCCCACAGAACATGCAGAAGGTCCAAGCTATTGGGTCTATCCGAGCGGCTATCGGAGAGAGTATGCTTGAAGGTTCCTCAATGGACCGCATTAAGCGAGTCACAGAAGGACAGTTCATTCGGGAAGCCAATATgg                                                                                                                                                                       <  1:492852/301‑1 (MQ=255)`  
                                                                                                                                   `ggCGCTCAGGCTAGCAGTGGTCAGAACGCTCAGGCCAAAATGATTGCCGCTCAGACCGCTGCTGGTCGTCGTCAAGCTATGGAAATCATGAGGCAGACGAACATCCAGAATGCTGACCTATCGTTGCAAGCTCGAAGTAAACTTGAGGAAGAGTCCGCCGAGTTGACCCCACAGAACATGCAGAAGGTCCAAGCTATTGGGTCTATCCGAGCGGCTATCGGAGAGAGTATGCTTGAAGGTTCCTCAATGGACCGCATTAAGCGAGTCACAGAAGGACAGTTCATTCGGGAAGCCAATATgg                                                                                                                                                                       <  1:348772/301‑1 (MQ=255)`  
                                                                                                                                           `ggCTATCAGTGGTCAGAACGCTCAGGCCAAAATGATTGCCGCTCAGACCGCTGCTGGTCGTCGTCAAGCTATGGAAATCATGAGGCAGACGAACATCCAGAATGCTGACCTATCGTTGCAAGCTCGAAGTAAACTTGAGGAAGCGTCCGCCGAGTTGACCCCACAGAACATGCAGAAGGTCCAAGCTATTGGGTCTATCCGAGCGGCTATCGGAGAGAGTATGCTTGAAGGTTCCTCAATGGACCGCATTAAGCGAGTCACAGAAGGACAGTTCATTCGGGAAGCCAATATGGAAACTgag                                                                                                                                                               >  1:219948/1‑301 (MQ=255)`  
                                                                                                                                              `tATCAGTGGTCAGAACGCTCAGGCCAAAATGATTGCCGCTCAGACCGCTGCTGGTCGTCGTCAAGCTATGGAAATCATGAGGCAGACGAACATCCAGAATGCTGACCTATCGTTGCAAGCTCGAAGTAAACTTGAGGAAGCGTCCGCCGAGTTGACCCCACAGAACATGCAGAAGGTCCAAGCTATTGGGTCTATCCGAGCGGCTATCAGAGAGAGTATGCTTGAAGGTTCCTCAATGGACCGCATTAAGCGAGTCACAGAAGGACAGTTCATTCGGGAAGCCAATATGGTAACTGAGAAc                                                                                                                                                            <  1:180149/301‑1 (MQ=255)`  
                                                                                                                                              `tATCAGTGGTCAGAACGCTCAGGACAAAATGATTGCCGCTCAGACCGCTGCTGGTCGTCGTCAAGCTATGGAAATCATGAGGCAGACGAACATCCAGAATGCTGACCTATCGTTGCAAGCTCGAAGTAAACTTGAGGAAGCGTCCGCCAAGTTGACCCCACAGAACATGCAGAAGGTCCAAGCTATTGGGTCTATCCGAGCGGCTATCGGAGAGAGTATGCTTGAAGGTTCCTCAATGGACCGCATTAAGCGAGTCACAGAAGGACAGTTCATTCGGGAAGCCAATATGGTAACTGAGAAc                                                                                                                                                            <  1:509288/301‑1 (MQ=255)`  
                                                                                                                                               `ataaGTGGTCAGAAAGCGCAGGCCAAAATGATTGCCGCTCAGACCGCTGCTGGTCGTCGTCAAGCTATGGAAATCATGAGGCAGACGAACATCCAGAATGCTGACCTATCGTTGCAAGCTCGAAGTAAACTTGAGGAAGCGTCCGCCGAGTTGACCCCACAGAACATGCAGAAGGTCCAAGCTATTGGGTCTATCCGAGCGGCTATCGGAGAGAGTATGCTTGAAGGTTCCTCAATGGACCGCATTAAGCGAGTCACAGAAGGACAGTTCATTCGGGAAGCCAATATGGTAACTGAGAACt                                                                                                                                                           <  1:337799/298‑1 (MQ=255)`  
                                                                                                                                                   `ggggTAAGAACGCTCAGGCCAAAATGATTGCCGCACAGACCGCTGCTGGTCGTCGTCAAGATATGGAAATCATGAGGCAGACGAACATCCAGAATGCTGACCTATCGTTGCAAGCTCGAAGTAAACTTGAGGAAGCGGCCGCCGAGTTGACCCCACAGAACATGCAGAAGGTCCTAGCTATTGGGTCTATCCGAGCGGCTATCGGAGAGAGTATGCTTGAAGGTTCCTCAATGGACCGCATTAAGCGAGTCACAGAAGGACAGTTCATTCGGGAAGCCAATATGGTAACTGAGAACTATCg                                                                                                                                                       <  1:503138/299‑1 (MQ=255)`  
                                                                                                                                                   `gTGGTCAGCACGCTCAGGCCAAAATGATTGCCGCTCAGACCGCTGCTGGTCGTCGTCAAGCTATGGAAATCATGAGGCAGACGAACATCCAGAATGCTGACCTATCGTTGCAAGCTCGAAGTAAACTTGAGGAAGCGTCCGCCGAGTTGACCCCACAGAACATGCAGAAGGTCCAAGCTGTTGGGTCTATCCGAGCGGCTATCGGAGAGAGTATGCTTGAAGGTTCCTCAATGGCCCACATTAAGCGAGTCACAGAAGGACAGTTCATTCGGGACGCCAATATggctgtctcttatacaca                                                                                                                                                       >  1:195991/1‑285 (MQ=255)`  
                                                                                                                                                              `gatCAGGCCAAAATGATTGCCGCGCAGACCGCTGCTGGTCGTCGTCAAGCTATGGAAATCATGAGGCAGACGAACATCCAGAATGCTGACCTATCGTTGCAAGCTCGAAGTAAACTTGAGGAAGCGTCCGCCGAGTTGACCCCACAGAACATGCAGAAGGTCCAAGCTATTGGGTCTATCCGAGCGGCTATCGGAGAGAGTATGCTTGAAGGTTCCTCAATGGACCGCATTAAGCGAGTCACAGAAGGACAGTTCATTCGGGAAGCCAATATGGTAACTGAGAACTATCGCCGTGACTAcc                                                                                                                                            <  1:343623/299‑1 (MQ=255)`  
                                                                                                                                                                    `gCCAAAATGATTGCTGCTCAGACCGCTGCTGGTCGTCGTCAAGCTATGGAAAGCATGAGGCAGACGAACATCCAGAATGCTGACCTATCGTTGCAAGCTCGAAGTAAACTTGAGGAATCGTCCGCCGAGTTGACCCCACAGAACATGCAGAAGGTCCAAGCTATTGGGTCTATCCGAGCGGCTATCGGAGAGAGTATGCTTGAAGGTTCCTCAATGGACCGCATTAAGCGAGTCACAGAAGGACAGTTCATTCGGGAAGCCAATATGGTAACTGAGAACTATCGACGTGACTACAAAGCaa                                                                                                                                      <  1:145079/301‑1 (MQ=255)`  
                                                                                                                                                                                    `cTCAGACCGCTGCTGGTCGTCGTCAAGCTATGGAAATCATGAGGCAGACGAACATCCAGAATGCTGACCTATCGTTGCAAGCTCGAAGTAAACTTGAGGAAGCGTCCGCCGAGTTGACCCCACAGAACATGCAGAAGGTCCAAGCTATTGGGTCTATCCGAGCGGCTATCGGAGAGAGTATGCTTGAAGGTTCATCAATGGACCGCATTAAGCGAGTAACAGAAGGACAGTTCATTCGGGAAGCCAATATGGTAACTGAGAACTATCGCCGTGACTACCAAGCAATCTTCGCACAGCAACt                                                                                                                      >  1:249917/1‑301 (MQ=255)`  
                                                                                                                                                                                          `ccGCTGCTGGTCGTCGTCAAGCTATGGAAATCATGAGGCAGACGAACATCCAGAATGCTGACCTATCGTTGCAAGCTCGAAGTAAACTTGAGGAAGCGTCCGCCGAGTTGACCCAACAGAACATGCAGAAGGTCCAAGCTATTGGGTCGATCCGGGCGGCTATCGGAGAGAATATGCTTGAAGGTTCCTCAATGGATCGCATTAAGCGAGTCACAGAAGGACAGTTCATTCGGGAAGCCAATATGGTAACTGAGAACTATCGCCGTGACTACAAAGCAATCTTCGCACAGCAACTtggtgg                                                                                                                <  1:639764/301‑1 (MQ=255)`  
                                                                                                                                                                                               `gctgGTCGTCGTCAAGCTATGGAAATCATGAGGCAGACGAACATCCAGAATGCTGACCTATCGTTGCAAGCTCGAAGTAAACTTGAGGAAGCGTCCGCCGAGTTGACCCCACAGAACATGCAGAAGGTCCAAGCTATTGGGTCTATCCGAGCGGCTATCGGAGAGAGTATGCTTGAAGGTTCCTCAATGGACCGCATTAAGCGAGTCACAGAAGGACAGTTCATTCGGGAAGCCAATATGGTAACTGAGAACTATCGCCGTGACTACCAAGCAATCTTCGCACAGCAACTTGGTGGTACTc                                                                                                           <  1:614489/301‑1 (MQ=255)`  
                                                                                                                                                                                                   `gtcgtcgtcAAGCTATGGAAATCATGAGGCAGACGAACATACAGAATGCTGACCTATCGTTGCAAGCTCGAAGTAAACTTGAGGAAGCGTCCGCCGAGTTGACCCCACAGAACATGCAGAAGGTCCAAGCTATTGGGTCTATCCGAGCGGCTATCGGAGAGAGTATGCTTGAAGGTTCCTCAATGGACCGCATTAAGCGAGTCACAGAAGGACAGTTCATTCGGGAAGCCAATATGGTAACTGAGAACTATCGCCGTGACTACCAAGCAATCTTCGCACAGCAACTTGGTGGTACTCAAAg                                                                                                       <  1:537209/301‑1 (MQ=255)`  
                                                                                                                                                                                                      `gtcgtcAAGCTATGGAAATCATGAGGCAGACGAATATCCAGAATGCTGACCTATCGTTGCAAGCTCGAAGTAAACTTGAGGAAGCGTCCGCCGAGTTGACCCCACAGAACATGCAGAAGGTTCAAGCTATTGGGTCTATCCGAGCGGCTATCGGAGAGAGTATGCTTGAAGGTTCCTCAATGGACCGCATTAAGCGAGTCACAGAAGGACAGTTCATTCGGGAAGCCAATATGGTAACTGAGAACTATCGCCGTGACTACCAAGCAATCTTCGCACAGCAACTTGGTGGTACTCAAAGtgc                                                                                                    <  1:302814/301‑1 (MQ=255)`  
                                                                                                                                                                                                       `tcgtcAAGCTATGGAAATCATGAGGCAGACGAACATCCAGAATGCTGACCTATCGTTGCAAGCTCGAAGTAAACTTGAGGAAGCGTCCGCCGAGTTGACCCCACAGAACATGCAGAAGGTCCAAGCTATTGGGTCTATCCGAGCGGCTATCGGAGATAGTATGCTTGAAGGTTCCTCAATGGACCGCATTAAGCGAGTCACCAAAGGACAGTTCATTCGGGAAGCCAATATGGTAACTGAGAACTATCGCCGTGACTACCAAGCAATCTTCGCACATCAACTTGGTGGTACTCAAAGtacg                                                                                                   >  1:632576/1‑298 (MQ=255)`  
                                                                                                                                                                                                                 `aTGGAAATCATGAGGCAGACGAACATCCAGAATGCTGACCTATCGTTGCAAGCTCGAAGTAAACTTGAGGAAGCGTCCGCCGAGTTGACCCCACAGAACATGCAGAAGGTCCAAGCTATTGGGTCTATCCGAGCGGCTATCGGATAGAGTATGCTTGAAGGTTCCTCAATGGACCGCATTAAGCGAGTCACCGAAGGACAGTTCATTCGGGAAGCCAATATGGTAACTGAGCACTATCGCCGTGCCTCCCAAGCAATCTTCGCACATCAACTTGATGGTACTCAAAGTGCTGCAATTCCGa                                                                                         >  1:320171/1‑301 (MQ=255)`  
                                                                                                                                                                                                                  `tGGAAATCATGAGGCAGACGAACATCCAGAATGCTGACCTATCGTTGCAAGCTCGAAGTAAACTTGAGGAAGCGTCCGCCGAGTTGACCCCACAGAACATGCAGAAGGTCCAAGCTATTGGGTCTATCCGAGCGGCTATCGGAGAGAGTATGCTTGAAGGTTCCTCAATGGACCGCATTAAGCGAGTCACAGAAGGACAGTTCATTCGGGAAGCCAATATGGTAACTGAGAACTATCGCCGTGACTACCAAGCAATCTTCGCACAGCAACTTGGTGGTACTcctgtctcttatacacatct                                                                                        >  1:607711/1‑282 (MQ=255)`  
                                                                                                                                                                                                                             `aGGCAGACGAACATCCAGAATGCTGACCTATCGTTGCAAGCTCGAAGTAAACTTGAGGAAGCGTCCGCCGAGTTGACCCCACAGAACATGCAGAAGGTCCAAGCTATTGGGTCTATCCGATCGGCTATCGGAGAGAGTATGCTTGAAGGTTCCTCAATGGACCGCATTAAGCGAGTCACAGAAGGACAGTTCATTCGGGAAGCCAATATGGTAACTGAGAACTATCTCCGTGACTACCAAGCAATCTTCGCACAGCAACTTGGTGGTACTCAAAGTGCTGCAAGTCAGATTGACGAAATCt                                                                             >  1:100153/1‑301 (MQ=255)`  
                                                                                                                                                                                                                                `cAGACGAACATCCAGAATGCTGACCTATCGTTGCAAGCTCGAAGTAAACTTGAGGAAGCGTCCGCCGAGTTGACCCCACAGAACATGCAGAAGGTCCAAGCTATTGGGTCTATCCGAGCGGCTATCGGAGAGAGTATGCTTGAAGGTTCCTCAATGGACCGCATTAAGCGAGTCACAGAAGGACAGTTCCTTCGGGAAGCCAATATGGTAACTGAGAACTATCGCCGTGACTACCAAGCAATCTTCGCACAGCAACTTGGTGGTACTCAAAGTGCTGCAAGTCAGAttctgtctcttatac                                                                          >  1:588177/1‑288 (MQ=255)`  
                                                                                                                                                                                                                                    `cGAACATCCAGAATGCTGACCTATCGTTGCAAGCTCGAAGTAAACTTGAGGAAGCGTCCGCCGAGTTGACCCCACAGAACATGCAGAAGGTCCAAGCTATTGGGTGTATCCGAGCGGCTATCGGAGAGAGTATGCTTGAAGGTTCCTCAATGGACCGCATTAAGCGAGTCACAGAAGGACAGTTCATTCGGGAAGCCAATATGGTAACTGAGAACTATCGCCGTGACTACCAAGCAATCTTCGCACAGCAACTTGGTGGTACTCAAAGTGCTGCAAGTCAGATTGACGAAATCTATAagag                                                                      <  1:11130/301‑1 (MQ=255)`  
                                                                                                                                                                                                                                        `cATCCAGAATGCTGACCTATCGTTGCAAGCTCGAAGTAAACTTGAGGAAGCGTCCGCCGAGTTGACCCCACAGAACATGCAGAAGGTACAAGCTATTGGGTCTATCCGAGCGGCTATCGGAGAGAGTATGCTTGAAGGTTTCTCAATGGAACGCATTAAGCGAGTCACAGAGGGACAGTTCATTCGGGAAGCCAAAATGGTAACTGAGAACTATCGCCGTGACTACCAAGCAATCTTTGCACAGCAACTTTGTGGTACTCAAAGTGCTGCAAGGCAGATTGACGAAATCTATAagagtcgt                                                                  >  1:105919/1‑297 (MQ=255)`  
                                                                                                                                                                                                                                         `aTCCAGAATGCTGACCTATCGTTGCAAGCTCGAAGTAAACTTGAGGAAGCGTCCGCCGAGTTGACCCCACAGAACATGCAGAAGGTCCAAGCTATTGGGTCTATCCGAGCGGCTATCGGAGAGAGTATGCTTGAAGGTTCCTCAATGGACCGCATTAAGCGAGTCACAGAAGGACAGTTCATTCGGGAAGCCAATATGGTAACTGAGAACTATCGCCGTGACTACCAAGCAATCTTCGCACAGCAACTTGGTGGTACTCAAAGTGCTGCAAGTCAGATTGACGAAATCTATAAGAGCGaac                                                                 >  1:406060/1‑301 (MQ=255)`  
                                                                                                                                                                                                                                           `ccAGAATGCTGACCTATCGTTGCAAGCTCGAAGTAAACTTGAGGAAGCGTCCGCCGAGTTGACCCCACAGAACATGCAGAAGGTCCAAGCTATTGGGTCTATCCGAGCGGCTATCGGAGAGAGTATGCTTGAAGGTTCCTCAATGGACCGCATTAAGCGAGTCACAGAAGGACAGTTCATTCGGGAAGCCAATATGGTAACTGAGAACTATCGCCGTGACTACCAAGCAATCTTCGCACAGCAACTTGGTGGTACTCAAAGTGCTGCAAGTCAGATTGACGAAATCTATAAGAGcctgtct                                                               >  1:281043/1‑295 (MQ=255)`  
                                                                                                                                                                                                                                              `gAATGCTGACCTATCGTTGCAAGCTCGAAGTAAACTTGAGGAAGCGTCCGCCGAGTTGACCCCACAGAACATGCAGAAGGTCCAAGCTATTGGGTCTATCCGAGCGGCTATCGGAGAGAGTATGCTTGAAGGTTCCTCAATGGACCGCATTAAGCGAGTCACAGAAGGACAGTTCATTCGGGAAGCCAATATGGTAACTGAGAACTATCGCCGTGACTACCAAGCAATCTTCGCACAGCAACTTGGTGGTACTCAAAGTGCTGCAAGTCAGATTGACGAAATCTATAAGAGCGaacagaaa                                                            >  1:202163/1‑301 (MQ=255)`  
                                                                                                                                                                                                                                               `aaTGCTGACCTATCGATGCAAGCTCGAAGTAAACTTGAGGAAGCGTCCGCCGAGTTGACCCCACAGAACATGCAGAAGGTCCAAGCTATTGGGTCTATCCGAGCGGCTATCGGAGAGAGTATGCTTGAAGGTTCCTCAATGGACCGCATTAAGCGAGTCACAGAAGGACAGTTCATTCGGGAAGCCAATATGGTAACTGAGAACTATCGCCGTGACTACCAAGCAATCTTCGCACAGCAACTTGGTGGTACTCAAAGTGCTGCAAGTCAGATTGACGAAATCTATAAGAGCGaacagaaac                                                           <  1:169247/301‑1 (MQ=255)`  
                                                                                                                                                                                                                                                    `ggACCTATCGTTGCAAGCTCGAAGTAAACTTGAGGAAGCGTCCGCCGAGTTGACCCCACAGAACATGCAGAAGGTCCAAGCTATTGGGTCTATCCGAGCGGCTATCGGAGAGAGTATGCTTGAAGGTTCCTCAATGGACCGCATTAAGCGAGTCACAGAAGTACAGTTCATTCGGGAAGCCAATATGGTAACTGAGAACTATCGCCGTGACTACCAAGCAATCTTCGCACAGCAACTTGGTGGTACTCAAAGTGCTGCAAGTCAGATTGACGAAATCTATAAGAGCGAACAGAAACagaag                                                      <  1:383960/300‑1 (MQ=255)`  
                                                                                                                                                                                                                                                         `tATCGTTGCAAGCTCGAAGTAACCTGGAGGAAGCGTCCGCCGAGTTGACCCCACAGAACATGCAGAAGGTCCAAGCTATTTGGTCTATCCGAGCGGCTATCGGAGAGAGTATGCTTGAAGGTCCCTCAATGGACCGCATTAAGCGAGTCACAGAAGGACAGTTCATTCGGGAAGCCAATATGGTAACTGAGAACTATCGCCGTGACTACCAAGCAATCTTCGCACAGCAACTTGGTGGTACTCAAAGTGCTGCAAGTCAGATTGACGAAATCTATAAGAGCGAACAGAAACAGAAGAGTaa                                                 <  1:82369/301‑1 (MQ=255)`  
                                                                                                                                                                                                                                                            `cgtggaaaGCTCGAAGTAAACTTGAGGAAGCGTCCGCCGAGTTGACCCCACAGAACATGCAGAAGGTCCAAGCTATTGGGTCTATCCGAGCGGCTATCGGAGAGAGTATGCTTGAAGGTTCCTCAATGGACCGCATTAAGCGAGTCACAGAAGGACAGTTCATTCGGGAAGCCAATATGGTAACTGAGAACTATCGCCGTGACTACCAAGCAATCTTCGCACAGCAACTTGGTGGTACTCAAAGTGCTGCAAGTCAGATTGACGAAATCTATAAGAGCGAACAGGAACAGAAGAGTAAGCt                                              <  1:64517/295‑1 (MQ=255)`  
                                                                                                                                                                                                                                                              `ttGCCAGGTCCAAGGAAACTTGAGGAAGCGTCCGCCCAGTTTAACCCACAGAACATGCAGAAGGTCCAAGCTATTGGGTCTATCCGAGCGGCTATCGGAGAGAGTATGCTTGAAGGTTCCTCAATGGACCGCATTAAGCGAGTCACAGAAGGACAGTTCATTCGGGAAGCCAATATGGTAACTGAGAACTATCGCCGTGACTACCAAGCAATCTTCGCACAGCAACTTGGTGGTACTCAAAGTGCTGCAAGTCAGATTGACGAAATCTATAAGAGCGAACAGAAACAGAAGAGTAAGCTAc                                            <  1:214142/301‑1 (MQ=255)`  
                                                                                                                                                                                                                                                                 `aaaGCTCGAAGTAAACTTGAGTAAGCGTCTGCCGAGTTGACCACACAGAACATGCAGAAGGTCCAAGGTATTGGCTATATCCGAGCGGCTAGCGGATAGAGTATGCTTGAAGGTTCCTCAATGGACCGAATTAAGCGAGTCACAGAAGGACAGTTCATTCGGGAAGCCAATATGGTAACTGAGAACTATCGCCGTGACTACCAAGCAATCTTCGCACAGCACCTTGGTGGTACTCAAAGTGCTGCAAGTCAGATTGCCGAAATCTATAAGAGCGAACAGAAACAGAAGAGTAAGCTACAGa                                         <  1:657190/300‑1 (MQ=255)`  
                                                                                                                                                                                                                                                                  `aaGGTCGAAGTAAACTTGAGGAAGCGTCCGCCGAGTTGAACCCACAGAACATGCAGAAGGTCCAAGCTATTGGGTCTATCCGAGCGGCTATCGGAGAGAGTATGCTTGAAGGTTCCTCAATGGACCGCATTAAGCGAGTCACAGAAGGACAGTTCATTCGGGAAGCCAATATGGTAACTGAGAACTATCGCCGTGACTACCAAGCAATCTTCGCACAGCAACTTGGTGGTACTCAAAGTGCTGCAAGTCAGATTGACGAAATCTATAAGAGCGAACAGAAACAGAAGAGTAAGCTACAGAt                                        <  1:74755/301‑1 (MQ=255)`  
                                                                                                                                                                                                                                                                  `aaGCTCGAAGTAAACTTGAGGAAGCGTCCGCCGAGTTGACCCCACAGAACATGCAGAAGGTCCAAGCTATTGGGTCTATCCGAGCGGCTATCGGAGAGAGTATGCTTGAATGTTCCTCAATGGACCGCATTAAGCGAGTCACAGAAGGACAGTTCATTCGGGAAGCCAATATGGTAACTGAGAACTATCGCCGTGACTACCAAGCAATCTTCGCACAGCAACTTGGTGGTACTCAAAGTGCTGCAAGTCAGATTGACGAAATCTATAAGAGCGAACAGAAACAGAAGAGTAAGCTACAGAt                                        <  1:411565/301‑1 (MQ=255)`  
                                                                                                                                                                                                                                                                     `cTCGAAGTAAACTTGAGGAAGCGTCCGCCGAGTTGACCCCACAGAACATGCAGAAGGTCCAAGCTATTGGGTATATCCGAGCGGCTATCGGAGAGAGTATGCTTGAAGGTTCCTCAATGGACCGCATTAAGCGAGTCACAGAAGGACAGTTCATTCGGGAAGCCAATATGGTAACTGAGAACTATCGCCGTGACTACCAAGCAATCTTCGCACAGCAACTTGGTGGTACTCAAAGTGCTGCAAGTCAGATTGACGAAATCTATAAGAGAGAACAGAAACAGAAGAGTAAGCTACAGATGGt                                     <  1:497958/301‑1 (MQ=255)`  
                                                                                                                                                                                                                                                                        `gAAGTAAACGTGAGGAAGCGTCCGCCGAGTTGACCCCACAGAACATGCAGAAGGTCCAAGCTATTGGGTCTATCCGAGCGGCTATCGGAGAGAGTATGCTTGAAGGTTCCTCAATGGACCGCATTAAGCGAGTCACGGAAGGACAGTTCATTCGGGAAGCCAATATGGTAACTGAGAACTATCGCCGTGACTACCAAGCAATCTTCGCACAGCAACTTGGTGGTACTCAAAGTGCTGCAAGTCAGATTGACGAAATCTATAAGAGCGAACAGAAACAGAAGAGTAAGCTACAGATGGTTCt                                  <  1:268214/301‑1 (MQ=255)`  
                                                                                                                                                                                                                                                                         `aaGTAAACTTGAGGAAGCGTCCGCCGAGTTGACCCCACAGAACATGCAGAAGGTCCAAGCTATTGGGTCTATCCGAGCGGCTATCGGAGAGAGTATGCTTGAAGGTTCCTCAATGGACCGCATTAAGCGAGTCACAGAAGGACAGTTCATTCGGGAAGCCAATATGGTAACTGAGAACTATCGCCGTGACTACCAAGCAATCTTCGCACAGCAACTTGGTGGTACTCAAAGTGCTGCAAGTCAGATTGACGAAATCTATAAGAGCGAACAGAAACAGAAGAGTAAGCTACAGATGGTTCTg                                 >  1:131972/1‑301 (MQ=255)`  
                                                                                                                                                                                                                                                                           `gaaaaatgggggAAGCGTGC‑CCGAGTTGAACCCACAGAACATGCAGAAGGTCAAAGCTAGTGGGTAGATCCGAGCGGATATCGGAGAGAGTATGCTTGAAGGTGCCTCAATGGACCGCATGAAGCGAGTCACAGAAGGACAGTTCATTCGGGAAGCCAATATGGTAACTGAGAACTATCGCCGTGACTACCAAGCAATCTTCGCACAGCAACTTGGTGGTACTCAAAGTGCTGCAAGTCAGATTGACGAAATCTATAAGAGCGAACAGAAACAGAAGAGTAAGCTACAGATGGTTCTGGAc                              <  1:661050/291‑1 (MQ=255)`  
                                                                                                                                                                                                                                                                           `gTAAACTTGAGGAAGCGTCCGCTGAGTTGACCCCACAGAACATGCAGAAGGTCCAAGCTATTGGGTCTATCCGAGCGGCTATCGGAGAGAGTATGCTTGAAGGTTCCTCAATGGACCGCATTAAGCGAGTCACAGAAGGACAGTTCATTCGGGAAGCCAATATGGTAACTGAGAACTATCGCCGTGACTACCAAGCAATCTTCGCACAGCAACTTGGTGGTACTCAAAGTGCTGCAAGTCAGATTGACGAAATCTATAAGAGCGAACAGACACAGAAGAGTAAGCTACAGATGGCTCTGGa                               >  1:65031/1‑301 (MQ=255)`  
                                                                                                                                                                                                                                                                            `tAAACTTGATGAAGCGTCCGCCGAGTTGACCCCACAGAACATGCAGAAGGTCCAAGCTATTGGGTCTATCCGAGCGGCTATCGGAGAGAGTATGCTTGAAGGTTCCTCAATGGACCGCATTAAGCGAGTCACAGAAGGACAGTTCATTCGGGAAGCCAATATGGTAACTGAGAACTATCGCCGTGACTACCAAGCAATCTTCGCACAGCAACTTGGTGGTACTCAAAGTGCTGCAAGTCAGATTGACGAAATCTATAAGAGCGAACAGAAACAGAAGAGTAAGCTACAGATGGTTCTGGAc                              <  1:869/301‑1 (MQ=255)`  
                                                                                                                                                                                                                                                                            `tAAAATTGAGGAAGCGTCCGCCGAGTTGACCCCACAGAACATGCAGAAGGTCCAAGCTATTGGGTCTATCCGAGAGGCTATCGGAGAGAGTATGCTTGAATGTTCCTCAATGGACCTCATTAAGCGAGTCACAGAAGGACAGTTCATTCGGGAAGCCAATATGGTAACTGAGAACTATCGCCGTGACTACCAAGCAATCTTCGCACAGCAACTTGGTGGTACTCAAAGTGCTGCAAGTCAGATTGACGAAATCTATAAGAGCGAACAGAAACAGAAGAGTAAGCTACAGATGGTTCTGGAc                              <  1:156054/301‑1 (MQ=255)`  
                                                                                                                                                                                                                                                                                `cTTGAGGAAGCGTCCGCCGAGTTGACCCCACAGAACATGCAGAAGGTCCAAGCTATTGGGTCTATCCGAGCGGCTATCGGAGAGAGTATGCTTGAAGGTTCCTCAATGGACCGCATTAAGCGAGTCACAGAAGGACAGTTCATTCGGGAAGCCAATATGGTAACTGAGAACTATCGCCGTGACTACCAAGCAATCTTCGCACAGCAACTTGGTGGTACTCAAAGTGCTGCAAGTCAGATTGACGAAATCTATAAGAGCGAACAGAAACAGAAGAGTAAGCTACAGAtctgtctcttataca                          >  1:307096/1‑287 (MQ=255)`  
                                                                                                                                                                                                                                                                                          `cGTCCGCCGAGTTGACCCCACAGAACATTCAGAAGGTCCAAGCTATTGGGTCTATCCGAGCGGCTATCGGAGAGAGTATGCTTGAAGGTTCCTCAATGGACCGCATTAAGCGAGTCACAGAAGGACAGTTCATTCGGGAAGCCAATATGGTAACTGAGAACTACCGCCGTGACTACCAAGCAATCTTCGCACAGCAACTTGGTGGTACTCAAAGTGCTGCAAGTCAGATTGACGAAATCTATAAGAGCGAACAGAAACAGAAGAGTAAGCTACAGATGGTTCTGGACCCACTGGCTATCAt                <  1:297393/301‑1 (MQ=255)`  
                                                                                                                                                                                                                                                                                          `cGTCCGCCGAGTTGACCCCACAGAACATGCAGAAGGTCCAAGCTATTGGGTCTATCCGAGCGGCTATCGGAGAGAGTATGCTTGAAGGTTCCTCAATGGACCGCATTAAGCGAGTCACAGAAGGACAGTTCATTCGGGAAGCCAATATGGTAACTGAGAACTATCGCCGTGACTACCAAGCAATCTTCGCACAGCAACTTGGTGGTACTCAAAGTGCTGCAAGTCAGATTGACGAAATCTATAAGAGCGAACAGAAACAGAAGAGTAAGCTACAGATGGTTCTGGACCCACTGGCTATCAt                <  1:389892/301‑1 (MQ=255)`  
                                                                                                                                                                                                                                                                                              `cgccgAGTTGACCCCACAGAACATGCAGAAGGTCCAAGCTATTGGGTCTATCCGAGCGGCTATCGGAGAGAGTATGCTTGAAGGTTCCTCAATGGACCGCATTAAGCGAGTCACAGAAGGACAGTTCATTCGGGAAGCCAATATGGTAACTGAGAACTATCGCCGTGACTACCAAGCAATCTTCGCACAGCAACTTGGTGGTACTCAAAGTGCTGCAAGTCAGATTGACGAAATCTATAAGAGCGAACAGAAACAGAAGAGTAAGCTACAGATGGTTCTGGACCCACTGGCTATCATgggg            <  1:189984/301‑1 (MQ=255)`  

                                                                                                                                                                                                                                                                                                           `|`                                                                                                                                                                                                                                                                                                           
`GACTATCGGTGCGGTATTCCATGAAGAGTACACACGAGATGGTCAATTTCAGTTATTTACAATCACGAAAGGAGGATAACCATATGTGTTGGGCAGCCGCAATACCTAT‑‑‑‑‑‑‑‑‑‑‑‑CGCTATATCTGGCGCTCAGGCTATCAGTGGTCAGAACGCTCAGGCCAAAATGATTGCCGCTCAGACCGCTGCTGGTCGTCGTCAAGCTATGGAAATCATGAGGCAGACGAACATCCAGAATGCTGACCTATCGTTGCAAGCTCGAAGTAAACTTGAGGAAGCGTCCGCCGAGTTGACCTCACAGAACATGCAGAAGGTCCAAGCTATTGGGTCTATCCGAGCGGCTATCGGAGAGAGTATGCTTGAAGGTTCCTCAATGGACCGCATTAAGCGAGTCACAGAAGGACAGTTCATTCGGGAAGCCAATATGGTAACTGAGAACTATCGCCGTGACTACCAAGCAATCTTCGCACAGCAACTTGGTGGTACTCAAAGTGCTGCAAGTCAGATTGACGAAATCTATAAGAGCGAACAGAAACAGAAGAGTAAGCTACAGATGGTTCTGGACCCACTGGCTATCATGGGGTCTTCCGCTG  >  NC_001604.1/27645‑28229`

  

Alignment Legend

Aligned base mismatch/match (shaded by quality score): `ATCG/ATCG` `< 3 ≤` `ATCG/ATCG` `< 10 ≤` `ATCG/ATCG` `< 22 ≤` `ATCG/ATCG` `< 34 ≤` `ATCG/ATCG` `< 38 ≤` `ATCG/ATCG`

Unaligned base: `atcg`    Masked matching base: `atcg`    Alignment gap: `‑`    Deleted base: `‑`
